# Supplementary material for: SARS-CoV-2 and other respiratory pathogens are detected in continuous air samples from congregate settings
Source: Nat Commun. 2022 Aug 11;13:4717. doi: 10.1038/s41467-022-32406-w (PMC9366802; doi:10.1038/s41467-022-32406-w)
Supplement: Supplementary file 1 — Supplementary Information [file 41467_2022_32406_MOESM1_ESM.pdf]

Supplementary Information

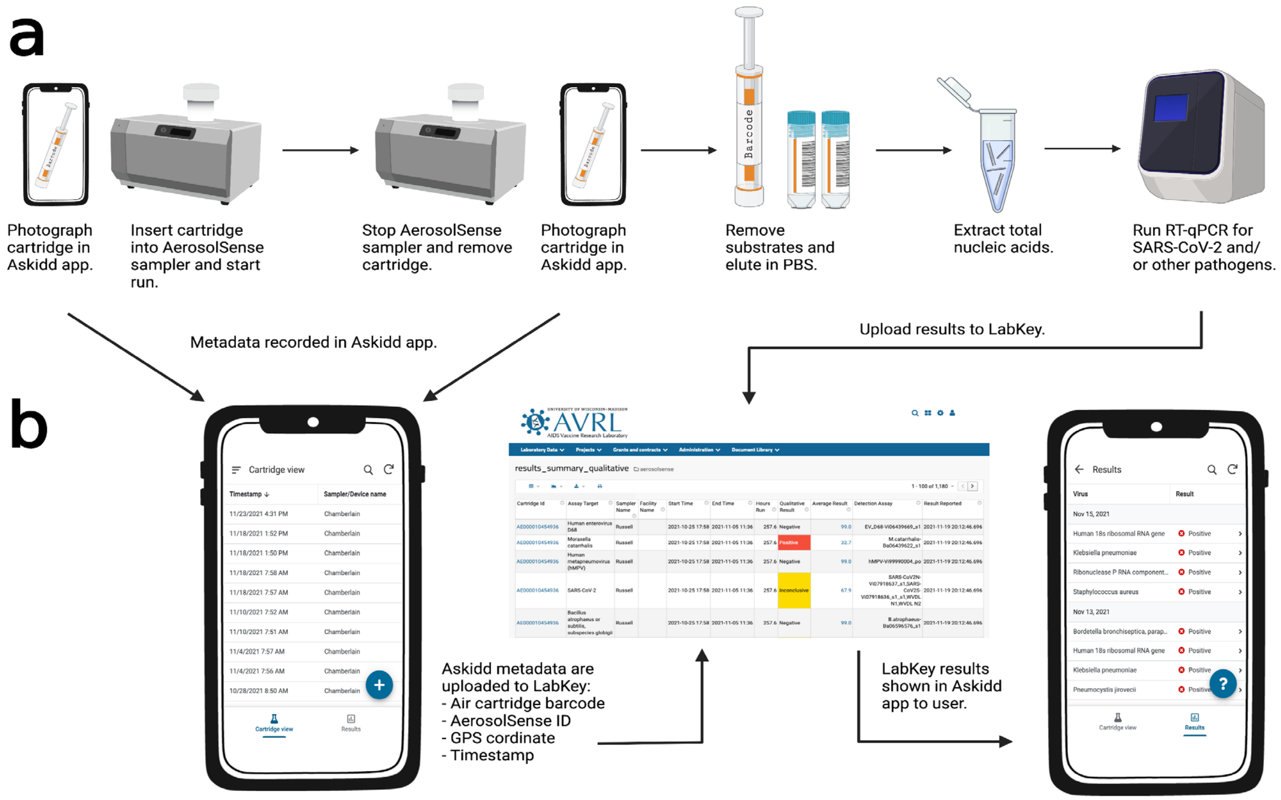

Supplementary Figure 01. Air sample testing workflow. (A) Overview of air sample collection, processing, and testing. (B) Air sample data collection and management. Individuals in charge of changing air cartridges at surveillance sites use the iOS and Android Askidd mobile app to collect metadata on air samples when cartridges are inserted and removed. Data are compiled in Labkey database and displayed to surveillance sites in the Askidd mobile app. Created with BioRender.com.

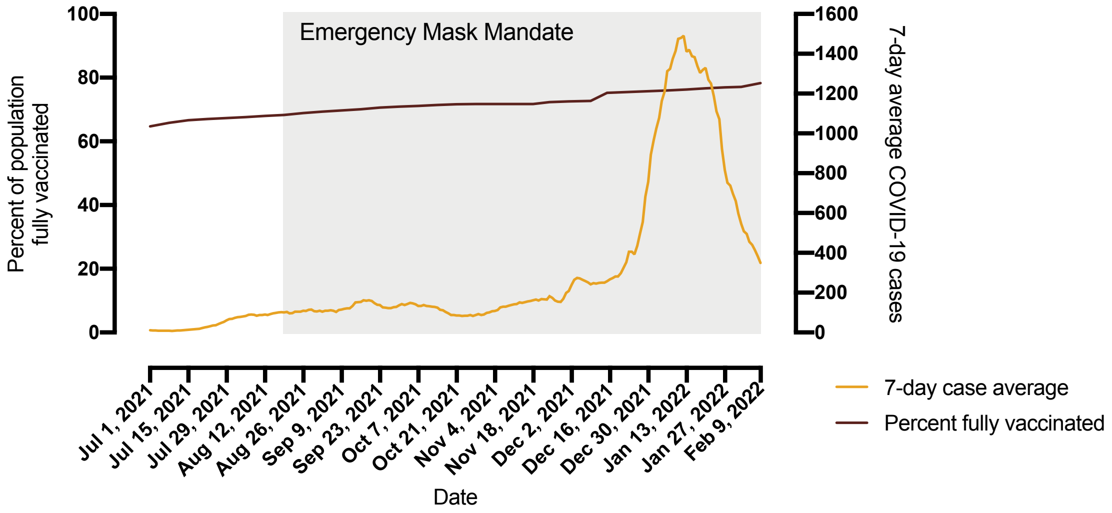

Supplementary Figure 02. COVID-19 seven-day average cases and vaccination rates in Dane County, WI. The percent of fully vaccinated individuals (red) is plotted on the left y-axis and seven-day average COVID-19 cases (orange) in Dane County, WI is plotted on the right y-axis with respect to time on the x-axis. Data were downloaded from Public Health Madison and Dane County (PHMDC) coronavirus dashboard (https://publichealthmdc.com/coronavirus/dashboard; accessed on March 3, 2022). Seven-day average COVID-19 cases were calculated from the sum of new cases per day for the most recent seven-day period divided by seven and rounded to the nearest whole number. The percent of the population fully vaccinated was calculated from the total number of residents who received all recommended doses in the primary series of the respective vaccines, this excludes additional recommended booster doses. The gray box shows the dates that a face-covering emergency order was implemented in Dane County, WI by PHMDC.

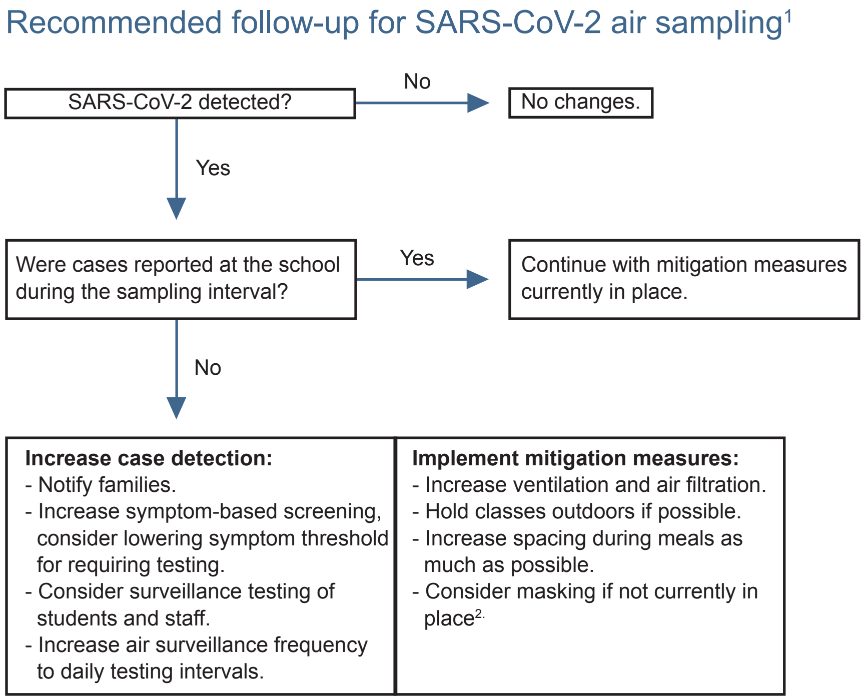

Supplementary Figure 03. SARS-CoV-2 air sampling response flowchart. Air sampling response decision flowchart was developed in collaboration with Public Health Madison and Dane County (PHMDC) to describe how schools may use air surveillance to implement risk mitigation strategies. [1] These recommendations supplement but would not supersede universal recommendations for all schools from PHMDC. [2] Universal masking recommended for all teachers, students, and staff in K-12 settings and may be required by public health orders.

Supplementary Table 1. TrueMark Respiratory Panel 2.0 TaqMan Array Card Crt cut-off values.

Target	Pathogen name (what is listed in Fig 4)	Minimum number of copies per reaction	Mean Crt	Comments
AdV_2of2-Vi99990002_po	Adenovirus	1.25	34.5	Adenovirus was called positive if AdV_1of2 or AdV_2of2 were positive.
B.pertussis-Ba06439623_s1	Bordetella spp.	1.25	34.63	Bordetella spp. was called positive if one of these targets were positive.
Bordetella-Ba06439624_s1	Bordetella spp.	1.25	31.93	
C.pneumoniae-Ba06439616_s1	Chlamydophila pneumoniae	50	33.41	
CoV_229E-Vi06439671_s1	Human coronavirus 299E	50	35.13	
CoV_NL63-Vi06439673_s1	Human coronavirus NL63	250	33.12	
CoV_OC43-Vi06439646_s1	Human coronavirus OC43	50	34.99	
EV_D68-Vi06439669_s1	Human enterovirus	1.25	33.11	Human enterovirus was called positive if EV_D6 or EV_pan were positive.
Flu_A_H1-Vi99990009_po	Influenza A virus	12.5	31.59	Influenza A virus was called positive if one of these targets were positive.
Flu_A_H3-Vi99990010_po	Influenza A virus	1.25	35.71	
Flu_A_pan-Vi99990011_po	Influenza A virus	1.25	33.89	
H.influenzae-Ba06439625_s1	Haemophilus influenzae	250	35.03	
HBoV-Vi99990003_po	Human bocavirus	12.5	32.58	
HHV3-Vi06439647_s1	Varicella zoster virus	12.5	35.7	
HHV4-Vi06439675_s1	Epstein-Barr virus	1.25	34.1	
HHV5-Vi06439643_s1	Cytomegalovirus	1.25	33.77	
hMPV-Vi99990004_po	Metapneumovirus	12.5	34.73	
hPIV1-Vi06439642_s1	Parainfluenza virus	50	33.94	Parainfluenza virus was called positive if one of these targets were positive.
hPIV2-Vi06439672_s1	Parainfluenza virus	12.5	34.08	
hPIV3-Vi06439670_s1	Parainfluenza virus	12.5	33.75	
hPIV4-Vi99990005_po	Parainfluenza virus	250	32.96	
K.pneumoniae-Ba04932083_s1	Klebsiella pneumoniae	1.25	34.16	
L.pneumophila-Ba06439617_s1	Legionella pneumophila	250	33.63	
M.catarrhalis-Ba06439622_s1	Moraxella catarrhalis	12.5	35.4	
M.pneumoniae-Ba06439620_s1	Myoplasma pneumoniae	12.5	27.97	
Measles -Vi99990013_po	Measles virus	1.25	35.3	
MERS_CoV-Vi06439644_s1	MERS	50	34.96	
Mumps-Vi06439657_s1	Mumps virus	12.5	30.01	
RSVA-Vi99990014_po	RSVA	12.5	36.58	
RSVB -Vi99990015_po	RSVB	12.5	33.61	
RV_1of2-Vi99990016_po	Human rhinovirus	50	35.35	Human rhinovirus was called positive if RV_1of2 or RV_2of2 were positive.
S.pneumoniae-Ba06439619_s1	Streptococcus pneumoae	250	33.67	
HPeV-Vi99990006_po	Human parechovirus	1.25	33.7	
Not detectable at less than 250 copies per reaction but included in Fig. 4 if Crt<30				
CoV_HKU1-Vi06439674_s1	Human coronavirus HKU1	NA	30	
EV_pan-Vi06439631_s1	Human enterovirus	NA	30	Human enterovirus was called positive if EV_D6 or EV_pan were positive.
Flu_B_pan-Vi99990012_po	Influenza B virus	NA	30	
RV_2of2-Vi99990017_po	Human rhinovirus	NA	30	Human rhinovirus was called positive if RV_1of2 or RV_2of2 were positive.
Detected in isolated pooled air cartridges but included in Fig. 4 if Crt<30				
AdV_1of2-Vi99990001_po	Adenovirus	NA	30	Adenovirus was called positive if AdV_1of2 or AdV_2of2 were positive
S.aureus-Ba04646259_s1	Staphylococcus aureus	NA	30	

Supplementary Table 2. Respiratory pathogens detected in air samples collected from congregate settings.

Category	Pathogen	Count
Acute Viruses	Adenovirus	5
	Human coronavirus OC43	7
	Influenza A virus	30
	Influenza A virus H3 subtype	19
	Human bocavirus	7
	Human parainfluenza virus (hPIV3)	1
	Respiratory syncytial virus A (RSVA)	2
	Respiratory syncytial virus B (RSVB)	1
Persistent Viruses	Epstein-Barr Virus (HHV4)	39
	Cytomegalovirus (HHV5)	35
Commensal Bacteria	Haemophilus influenzae	1
	Klebsiella pneumoniae	91
	Moraxella catarrhalis	26
	Staphylococcus aureus	89
	Streptococcus pneumoniae	5
Atypical bacteria	Bordetella spp.	1

Supplementary Table 3. Primer sequences used to amplify SARS-CoV-2 spike RBD from Gregory et al. 2021.

Primer Name	Primer Sequences 5’ -> 3’
Primary PCR forward	CTGCTTTACTAATGTCTATGCAGATTC
Primary PCR reverse	TCCTGATAAAGAACAGCAACCT
Secondary PCR forward	acactctttccctacacgacgctcttccgatctGTGATGAAGTCAGACAAATCGC
Secondary PCR reverse	gtgactggagttcagacgtgtgctcttccgatctATGTCAAGAATCTCAAGTGTCTG

Upper-case letters target the SARS-CoV-2 RBD and lower-case letters represent adapter sequence.
