# Supplementary material for: SARS-CoV-2 and other respiratory pathogens are detected in continuous air samples from congregate settings
Source: Nat Commun. 2022 Aug 11;13:4717. doi: 10.1038/s41467-022-32406-w (PMC9366802; doi:10.1038/s41467-022-32406-w)
Supplement: Supplementary file 3 — Description of Additional Supplementary Files [file 41467_2022_32406_MOESM3_ESM.pdf]

**Description of Additional Supplementary Files**

File Name: Supplementary Data 1
Description: Air sample metadata and SARS-CoV-2 RT-qPCR results.

File Name: Supplementary Data 2
Description: Air sample metadata and TrueMark results.
