# Supplementary material for: SARS-CoV-2 and other respiratory pathogens are detected in continuous air samples from congregate settings
Source: Nat Commun. 2022 Aug 11;13:4717. doi: 10.1038/s41467-022-32406-w (PMC9366802; doi:10.1038/s41467-022-32406-w)
Supplement: Supplementary file 6 — Reporting Summary [file 41467_2022_32406_MOESM6_ESM.pdf]

nature portfolio

Corresponding author(s): Shelby O'Connor

Last updated by author(s): 2022-7-11

Reporting Summary

Nature Portfolio wishes to improve the reproducibility of the work that we publish. This form provides structure for consistency and transparency in reporting. For further information on Nature Portfolio policies, see our Editorial Policies and the Editorial Policy Checklist.

Statistics

For all statistical analyses, confirm that the following items are present in the figure legend, table legend, main text, or Methods section.

n/a	Confirmed	
☐	☒	The exact sample size (n) for each experimental group/condition, given as a discrete number and unit of measurement
☐	☒	A statement on whether measurements were taken from distinct samples or whether the same sample was measured repeatedly
☒	☐	The statistical test(s) used AND whether they are one- or two-sided<br>*Only common tests should be described solely by name; describe more complex techniques in the Methods section.*
☒	☐	A description of all covariates tested
☒	☐	A description of any assumptions or corrections, such as tests of normality and adjustment for multiple comparisons
☐	☒	A full description of the statistical parameters including central tendency (e.g. means) or other basic estimates (e.g. regression coefficient) AND variation (e.g. standard deviation) or associated estimates of uncertainty (e.g. confidence intervals)
☒	☐	For null hypothesis testing, the test statistic (e.g. F, t, r) with confidence intervals, effect sizes, degrees of freedom and P value noted<br>*Give P values as exact values whenever suitable.*
☒	☐	For Bayesian analysis, information on the choice of priors and Markov chain Monte Carlo settings
☒	☐	For hierarchical and complex designs, identification of the appropriate level for tests and full reporting of outcomes
☒	☐	Estimates of effect sizes (e.g. Cohen's d, Pearson's r), indicating how they were calculated

Our web collection on statistics for biologists contains articles on many of the points above.

Software and code

Policy information about availability of computer code

Data collection

We gathered air sample metadata using the IOS and Android Askidd mobile app and LabKey database. We generated RT-qPCR and TMA data for SARS-CoV-2 and other respiratory pathogens using the QuantStudio 7 Pro Real-Time PCR System, Hologic Pather System, QuantStudio 3 Real-Time PCR System, and Applied Biosystems COVID-19 Interpretive Software.

Data analysis

TrueMark analysis:
We generated TrueMark cycle relative threshold values for air samples using the Design and Analysis v2.6.0 software from Thermo Fisher Scientific, Inc. using the suggested parameters from the manufacturer described in the TrueMark respiratory panel 2.0 manual. TrueMark results were exported from the quality check module in the Design and Analysis Software v2.6.0 as a CSV. Analysis was performed using a custom R script (v. 3.6.0) in RStudio (v. 1.3.959) to filter amplified results using the following cut-off values: amplification score >1.2 and Crt confidence >0.7. Samples were further filtered on pathogen-specific Crt cut-off values determined in a limit-of-detection experiment (Supplementary Data 1).
SARS-CoV-2 RBD Sequencing analysis:
The data were analyzed using a custom workflow implemented in Snakemake v7.3.4 (https://snakemake.github.io/) and is publicly available at https://github.com/dholab/SARS-CoV-2-Spike-RBD-Analysis. Briefly, paired-end reads were interleaved and merged into synthetic reads spanning the entire RBD PCR amplicon using bbmerge.sh (v38.93) from the bbtools package (sourceforge.net/projects/bbmap/) with default parameters. The merged reads were mapped to the SARS-CoV-2 reference sequence (Genbank MN908947.3) using minimap2 (v2.24) with the `-ax sr` preset for short reads. The resulting mapping file was sorted with samtools (v1.14). Reads that fully contain the desired amplicon sequence were extracted with the bedtools (v2.30.0) intersect tool. These reads were then downsampled to a target depth of 1000 reads using reformat.sh (v38.93) from the bbtools package. These downsampled reads were remapped to the MN908947.3 reference with minimap2. Residual PCR primer sequences were then trimmed with samtools ampliconclip using the `--hard-clip --both-ends` parameters. Next, a consensus sequence was generated by first generating a pileup with the samtools mpileup tool using default settings and then generating a consensus with ivar (v1.3.1) using the parameters `-q 20 -t 0 -m 20`.
At the same time a consensus sequence was generated, the primer-trimmed reads were deduplicated to determine how many of the reads were identical, essentially defining pseudo-haplotypes. Vsearch (v2.21.1) fastx_uniques tool was used for deduplicating and enumerating the

number of identical reads in each sample. Lineage-defining mutations in the RBD were used to differentiate Delta from Omicron consensus sequences.
SARS-CoV-2 whole genome sequencing analysis:
Raw sequencing reads generated using the Integrated DNA Technologies ARCTIC V4 primer panel were analyzed using the Illumina® DRAGEN COVID Lineage App, which uses a customized version of the DRAGEN DNA pipeline to perform Kmer-based detection of SARS-CoV-2. The app aligns reads to a reference genome, calls variants, and generates a consensus genome sequence. Lineage/clade assignments were also confirmed using NextClade (https://clades.nextstrain.org/, version 1.14.0) and Pangolin COVID-19 Lineage Assigner (https://pangolin.cog-uk.io/, version 3.1.20) by uploading obtained FASTA files.

For manuscripts utilizing custom algorithms or software that are central to the research but not yet described in published literature, software must be made available to editors and reviewers. We strongly encourage code deposition in a community repository (e.g. GitHub). See the Nature Portfolio guidelines for submitting code & software for further information.

Data

Policy information about availability of data

All manuscripts must include a data availability statement. This statement should provide the following information, where applicable:

- Accession codes, unique identifiers, or web links for publicly available datasets
- A description of any restrictions on data availability
- For clinical datasets or third party data, please ensure that the statement adheres to our policy

The SARS-CoV-2 sequencing data generated in this study have been deposited in the Sequence Read Archive (SRA) under bioprojects PRJNA811594 [https://www.ncbi.nlm.nih.gov/bioproject/PRJNA811594] and PRJNA856293. Consensus sequences generated from environmental samples with ARCTIC V4 were shared publicly on Global Initiative on Sharing All Influenza Data (GISAID) (www.gisaid.org) (EPI_ISL_8879388 and EPI_ISL_8879389). Air sample metadata and SARS-CoV-2 RT-qPCR are provided in Supplementary Data 1. The truemark air sample data generated in this study are provided in Supplementary Data 2. We obtained county-wide COVID-19 case data for Dane County from Public Health Madison and Dane County COVID-19 Dashboard (https://publichealthmdc.com/coronavirus/dashboard).

Field-specific reporting

Please select the one below that is the best fit for your research. If you are not sure, read the appropriate sections before making your selection.

☐ Life sciences ☐ Behavioural & social sciences ☒ Ecological, evolutionary & environmental sciences

For a reference copy of the document with all sections, see nature.com/documents/nr-reporting-summary-flat.pdf

Ecological, evolutionary & environmental sciences study design

All studies must disclose on these points even when the disclosure is negative.

Study description	The work in this study describes the collection and analysis of air samples for detection of genetic material derived from SARS-CoV-2 and other respiratory pathogens. Air samples were collected from indoor congregate settings using active air samplers. Nucleic acids were isolated from air cartridge substrates for PCR testing and genomic sequencing.
Research sample	Air samples collected at daily and weekly sampling intervals from 15 different congregate settings across communities in the Upper Midwestern states of Wisconsin and Minnesota. Air samples contain a mixture of exhaled components from many individuals and can capture pathogen-containing droplets and aerosols from infectious individuals, enabling virus detection independent of symptoms, test-seeking behavior, and access to swab-based testing. Air samples represent an efficient pooled sample that can provide information on asymptomatic and symptomatic SARS-CoV-2 infections.
Sampling strategy	Air samples were collected using AerosolSense Samplers (Thermo Fisher Scientific, Inc.) from each of the 15 congregate settings. We did not predetermine sample size. Sample size was determined by the established collection period July 2021 to February 2022. We included all available air samples collected from congregate settings that passed testing quality control parameters during this time.
Data collection	Air samples were collected by personnel from each of the 15 congregate settings, air sample metadata and testing data were gathered in a centralized location using a workflow relying on IOS and Android Askidd mobile app and LabKey database. Air sampler users open the Askidd app, and take a picture of the air cartridge barcode when installed and removed from the machine. The Askidd app collects GPS coordinates of the air sampler, timestamp, AerosolSense instrument ID, and air cartridge barcode to send to LabKey. When air sample testing was completed in the lab the results were uploaded to the Labkey database and displayed in the Askidd mobile app with associated metadata.
Timing and spatial scale	The data presented in this study was collected between July 19, 2021 and February 9, 2022. Air samples were collected from 15 congregate spaces from the Upper Midwestern states of Wisconsin and Minnesota.
Data exclusions	No data were excluded here. We included all RT-qPCR results generated from samples with no observed contamination in experimental controls. We also included all consensus sequences generated from the established sampling period.
Reproducibility	Throughout the study, SARS-CoV-2 RT-qPCR was performed on samples using multiple gene targets, samples were considered positive if two or more targets showed amplification. Both Sanger and MiSeq sequencing was performed on SARS-CoV-2 spike RBD, only MiSeq data is described in the manuscript and deposited in SRA. Whole genome sequencing was successfully performed on air

	samples in separate labs using the Illumina ARCTIC V4 protocol and Oxford Nanopore Technologies Midnight protocol, only ARCTIC V4 data is described in the manuscript and deposited in SRA.
Randomization	Experimental randomization was not applicable to this study. Air samples were grouped by collection date and geolocation.
Blinding	Air samples collected in Dane County were coded, RT-qPCR was performed blinded. Air is collected from the environment, so that no individual person is represented.

Did the study involve field work? ☐ Yes ☒ No

Reporting for specific materials, systems and methods

We require information from authors about some types of materials, experimental systems and methods used in many studies. Here, indicate whether each material, system or method listed is relevant to your study. If you are not sure if a list item applies to your research, read the appropriate section before selecting a response.

Materials & experimental systems

n/a	Involved in the study	
☒	☐	Antibodies
☒	☐	Eukaryotic cell lines
☒	☐	Palaeontology and archaeology
☒	☐	Animals and other organisms
☒	☐	Human research participants
☒	☐	Clinical data
☒	☐	Dual use research of concern

Methods

n/a	Involved in the study	
☒	☐	ChIP-seq
☒	☐	Flow cytometry
☒	☐	MRI-based neuroimaging
